# Methylation of leukocyte DNA and ovarian cancer: relationships with disease status and outcome

**DOI:** 10.1186/1755-8794-7-21

**Published:** 2014-04-28

**Authors:** Brooke L Fridley, Sebastian M Armasu, Mine S Cicek, Melissa C Larson, Chen Wang, Stacey J Winham, Kimberly R Kalli, Devin C Koestler, David N Rider, Viji Shridhar, Janet E Olson, Julie M Cunningham, Ellen L Goode

**Affiliations:** 1 Department of Biostatistics, University of Kansas, Medical Center, 3901 Rainbow Blvd, Kansas City, KS 66160, USA; 2 Department of Health Sciences Research, Mayo Clinic College of Medicine, Rochester, MN 55905, USA; 3 Department of Medicine, Mayo Clinic College of Medicine, Rochester, MN 55905, USA; 4 Department of Community and Family Medicine, Geisel School of Medicine at Dartmouth College, Lebanon, NH 03756, USA; 5 Department of Laboratory Medicine and Pathology, Mayo Clinic College of Medicine, Rochester, MN 55905, USA

**Keywords:** DNA methylation, CpG genotyping arrays, Epithelial ovarian cancer, Pathway, Etiology, Overall survival

## Abstract

**Background:**

Genome-wide interrogation of DNA methylation (DNAm) in blood-derived leukocytes has become feasible with the advent of CpG genotyping arrays. In epithelial ovarian cancer (EOC), one report found substantial DNAm differences between cases and controls; however, many of these disease-associated CpGs were attributed to differences in white blood cell type distributions.

**Methods:**

We examined blood-based DNAm in 336 EOC cases and 398 controls; we included only high-quality CpG loci that did not show evidence of association with white blood cell type distributions to evaluate association with case status and overall survival.

**Results:**

Of 13,816 CpGs, no significant associations were observed with survival, although eight CpGs associated with survival at p < 10^-3^, including methylation within a CpG island located in the promoter region of *GABRE* (p = 5.38 x 10^-5^, HR = 0.95). In contrast, 53 CpG methylation sites were significantly associated with EOC risk (p <5 x10^-6^). The top association was observed for the methylation probe cg04834572 located approximately 315 kb upstream of *DUSP13* (p = 1.6 x10^-14^). Other disease-associated CpGs included those near or within *HHIP* (cg14580567; p =5.6x10^-11^), *HDAC3* (cg10414058; p = 6.3x10^-12^), and *SCR* (cg05498681; p = 4.8x10^-7^).

**Conclusions:**

We have identified several CpGs in leukocytes that are differentially methylated by case-control status. Since a retrospective study design was used, we cannot differentiate whether DNAm was etiologic or resulting from EOC; thus, prospective studies of EOC-associated loci are the critical next step.

## Background

The role of DNA methylation (DNAm) in ovarian cancer is multi-faceted. While tumor tissue shows clear methylation patterns associating with histopathology, the role of blood-based DNAm patterns on disease etiology and outcome has been a subject of growing interest [1-4]. This includes study of variation in inherent global methylation levels, the relationship between exogenous exposures and leukocyte methylation, and the role of inherited variants on leukocyte methylation (mQTL) [5-8]. Five of the eleven confirmed ovarian cancer susceptibility variants and an endometriosis locus are located in homeobox gene clusters (*HOXA*, *HOXB*, and *HOXD*), homeobox related genes (*HNF1B*), or genes expressed in early progenitor cells (*TERT*) [9-13]. Thus, we hypothesize that DNAm levels in circulating systemic leukocytes of ovarian cancer cases and controls may differ, and that among cases, leukocyte methylation may vary by disease outcome.

Previous work by Teschendorff et al. (2009) [14] identified peripheral blood methylation signatures that predicted ovarian cancer case-control status using methylation measurements at more than 27,000 CpGs in 113 cases and 148 controls. However, as pointed out in the discussion by Teschendorff et al. and subsequently by Koestler et al.(2009) [15] and Houseman et al. (2012) [16], blood-based methylation measurements are dependent on distribution of white blood cell (leukocyte) types and the distribution of cell types is also related to disease status (i.e., confounding). Therefore, in order to minimize confounding by distribution of cell types, we performed case-control and survival analyses using 336 EOC cases and 398 controls, accounting for cell type associations to better understand the role of blood-based DNAm in ovarian cancer risk and survival.

## Methods

### Study participants

Eligible EOC cases were women aged 20 years or above who were ascertained between 2000 and 2009 at the Mayo Clinic within one year of diagnosis with pathologically confirmed primary epithelial ovarian, fallopian tube, or primary peritoneal cancer. Controls were recruited from among women seen at the Mayo Clinic for general medical examinations and individually-matched to cases on age (1-year) and area of residence. Women were of European descent and residing in a six-state area surrounding Minnesota, representing >85% of EOC cases seen at the Mayo Clinic, and cases had not begun chemotherapeutic treatment prior to blood draw. Table 1 summarizes characteristics of 734 participants, following quality control as outlined below. Peripheral blood (leukocytes) was used as the source of DNA, which was extracted from 10 to 15 mL of fresh peripheral blood by the Gentra AutoPure LSPuregene salting out methodology (Gentra) and stored at -80°C. Samples were bar-coded to ensure accurate processing. This work was approved by the Mayo Clinic Institutional Review Boards and all participants provided written informed consent.

**Table 1:** Characteristics of study participants

**Variable**	Batch 1*	Batch 2*	**Batch 3^**	**Total**
**Age at case diagnosis**				
N	69	146	121	336
Mean (SD)	60.2 (12.0)	63.3 (12.8)	62.2 (11.3)	62.3 (12.1)
Range	(33.0-82.0)	(28.0-91.0)	(33.0-86.0)	(28.0-91.0)
**Age at control enrollment**				
N	87	176	135	398
Mean (SD)	60.2 (12.1)	62.9 (12.7)	62.4 (11.4)	62.2 (12.2)
Range	(33.0-85.0)	(27.0-89.0)	(33.0-88.0)	(27.0-89.0)
**EOC case vital status**				
Alive	35 (51%)	51 (35%)	59 (49%)	145 (43%)
Deceased	34 (49%)	95 (65%)	62 (51%)	191 (57%)
**Follow-up time (years)**				
Mean (SD)	2.8 (1.5)	4.1 (2.8)	3.0 (2.5)	3.4 (2.5)
Range	(0.1-6.4)	(0.0-11.0)	(0.1-11.4)	(0.0-11.4)
**Parity**				
Nulliparous	25 (16%)	47 (15%)	34 (13%)	106 (14%)
1-2	49 (32%)	104 (32%)	109 (43%)	262 (36%)
3+	77 (50%)	156 (48%)	97 (38%)	331 (45%)
Unknown	4 (2%)	15 (5%)	16 (6%)	35 (5%)
**Smoking status**				
Never/former	140 (90%)	284 (88%)	213 (83%)	637 (87%)
Current	10 (6%)	19 (6%)	20 (8%)	49 (7%)
Unknown	6 (4%)	19 (6%)	23 (9%)	48 (7%)
**Alcohol use**				
Current	93 (60%)	172 (53%)	133 (52%)	398 (54%)
Former	22 (14%)	50 (16%)	47 (18%)	119 (16%)
Never	34 (22%)	73 (23%)	48 (19%)	155 (21%)
Unknown	7 (5%)	27 (8%)	28 (11%)	62 (8%)
**Histology, cases only**				
Serous	46 (66.7%)	97 (66.4%)	100 (82.6%)	243 (72%)
Endometrioid	16 (23.2%)	32 (22%)	15 (12%)	63 (19%)
Clear cell	4 (5.8%)	9 (6%)	3 (3%)	16 (5%)
Mucinous	1 (1.4%)	4 (3%)	3 (3%)	8 (2%)
Other	2 (2.9%)	4 (3%)	0 (0%)	6 (2%)
**Grade, cases only**				
Grade 1 or 2	11 (16%)	29 (20%)	16 (13%)	56 (17%)
Grade 3 or 4	56 (81%)	117 (80%)	103 (85%)	276 (82%)
Unknown	2 (3%)	0 (0%)	2 (2%)	4 (1%)
**Stage, cases only**				
Stage I or II	14 (20%)	39 (27%)	21 (17%)	74 (22%)
Stage III or IV	55 (80%)	107 (73%)	100 (83%)	262 (78%)
**Surgical debulking, cases only**				
Optimal (<1 cm)	57 (83%)	123 (84%)	105 (87%)	285 (85%)
Sub-optimal (>1 cm)	11 (16%)	22 (15%)	14 (12%)	47 (14%)
Unknown	1 (1%)	1 (1%)	2 (2%)	4 (1%)
**Ascites, cases only**				
No	39 (57%)	65 (45%)	76 (63%)	180 (54%)
Yes	23 (33%)	42 (29%)	32 (26%)	97 (29%)
Unknown	7 (10%)	39 (27%)	13 (11%)	59 (18%)

*Assayed using the Illumina Infinium HumanMethylation27 BeadChip.

^Assayed using the Illumina Infinium HumanMethylation450 BeadChip.

### DNA methylation assays and arrays

Peripheral blood (leukocytes) was used as the source of DNA. DNA was extracted from four milliliters of fresh peripheral blood using the Autogen Flexstar instrument utilizing Flexigene chemistry (salting out methodology). Blood is aliquoted for DNA extraction using an automated liquid handler with barcoding to ensure proper sample placement. Post-DNA extraction, DNA is aliquoted into a permanent storage tube utilizing an automated liquid handler with barcoding, again, to ensure proper sample placement. DNA samples are assessed for quality and concentration using a Trinean DropSense 96 spectrophotometer and DNA is then stored longterm at -80°C. The leukocyte-derived DNA (1 ug) was bisulfite modified (BSM) using the Zymo EZ96 DNA Methylation Kit (Zymo Research, Orange, CA) according to the manufacturer’s protocol. BSM DNA (250 ng) was then assayed on 96 well plates in three batches at the Mayo Clinic Molecular Genome Facility (Rochester, MN): Batch 1 used the Infinium HumanMethylation27 BeadChip on 84 cases and 91 controls, Batch 2 used this array on 172 cases and 176 controls and Batch 3 used Illumina Infinium HumanMethylation450 BeadChip on 156 cases and 157 controls. Methylation status at the target CpG sites was determined by comparing the ratio of fluorescent signal from the methylated allele to the sum from the fluorescent signal from both methylated and unmethylated alleles (i.e., the beta value).

To assess the quality of the DNAm data produced from the Illumina arrays, Centre d'Etudes du Polymorphisme Humain (CEPH) DNA, positive BSM controls (placental DNA) and negative BSM controls (whole genome amplified [WGA] DNA) were assayed within each batch. For the HumanMethylation27 BeadChips (Batch 1 and Batch 2), 9 CEPH DNA, 12 positive control DNA samples and 8 negative control DNA samples were also assayed, in addition to 12 replicate/duplicate samples. Similarly, for the HumanMethylation450 BeadChip batch (Batch 3), 6 CEPH samples, 11 positive control samples, 6 negative control samples and 6 replicate samples were assayed. Lastly, twenty duplicate samples were assayed using both Illumina Infinium HumanMethylation27 and HumanMethylation450 BeadChip in order to compare the methylation levels between the two arrays.

### Quality control and normalization

Using Illumina GenomeStudio software, DNAm values from the HumanMethylation27 BeadChip assays were scored as beta values, ranging from 0 (unmethylated) to 1 (methylated), resulting in methylation beta values for 27,578 probes. Quality control was done for Batch 1 and Batch 2 combined and then separately for Batch 3. Probes were then excluded if they were on the Y chromosome, positioned at a single nucleotide polymorphism (dbSNP build 137), had high beta values in BSM negative controls (beyond four standard deviations of mean), or were detected in less than 70% of samples. Quality control was also conducted at the sample level, based on the bisulfite conversion ratio and call rate rates (based on a detection p-value of 0.05). Histograms and scatterplots of these statistics were used to determine which samples to exclude (i.e., “outliers”). Similar quality control steps were completed for the samples assayed using the HumanMethylation450 BeadChips, which contained 485,577 CpG site-specific probes.

For the HumanMethylation27 BeadChip arrays, 25,922 (94%) methylation probes passed quality control; for the HumanMethylation450, 441,716 (91%) methylation probes passed quality control. The pairwise correlations for beta values among CEPH replicates were excellent (≥0.97 for Batches 1 and 2, and >0.99 for Batch 3), as were the intra-class correlations of beta values among CEPH replicates (>0.98 for Batches 1 and 2, and >0.99 for Batch 3) and among duplicated study participant samples (>0.93 for Batches 1 and 2, and >0.81 for Batch 3). For 20 samples assessed across batches, the intra-class correlation for beta values of the 24,520 overlapping probes in the HumanMethylation27 and HumanMethylation450 BeadChips was > 0.88. Of samples in Batches 1 and 2, 6 were excluded based on call rates, and one failed bisulfite conversion; in Batch 3, 10 samples were removed following quality control (9 samples failed the bisulfite conversion, one sample with low mean methylation beta value across probes). Following exclusions, we included 69 cases and 87 controls in Batch 1, 146 cases and 176 controls in Batch 2, and 121 cases and 135 controls in Batch 3.

We assessed possible differences by plate and chips within plates (8 BeadChips per plate were assessed with 12 DNAs each) through principal component analyses. Based on the assessment of technical artifacts using principal component analyses, a plate effect was observed within each of the three batches and a chip within batch effect for the HumanMethylation27 data (Additional file 1: Figure S1). Therefore, we adjusted for a plate effect for batch 3 and for chip within plate effect for batches 1 and 2 using a linear model of the logit-transformed beta value for each CpG site, with the unstandardized residuals saved. The logit-transformed locus mean was added back onto the residuals followed by the transformation of the residual to the 0 to 1 scale, producing an “adjusted beta” value for all CpG sites.

Finally, we restricted analyses to probes in common between the DNAm arrays following quality control, excluding 9,341 CpG probes on the Illumina Infinium HumanMethylation27 shown to associate with cell type distribution at q-value < 0.05 [15,16], as well as 1,363 CpG probes found by Chen et al. to be non-specific (i.e., mapped to multiple places along the genome) [17]. Thus, analyses focused on the remaining 13,816 CpG probes (i.e., 24,520 probes in common between the two panels following quality control minus 9,341 probes associated with cell type distribution minus 1,363 non-specific probes).

### Statistical association analysis

We analyzed each batch separately using Van der Waerden rank, or rank-based inverse Gaussian, transformed beta values and combined results across batch using meta-analysis techniques. This allowed us to examine similarity of effects across batches and to estimate the combined effect. Meta-analysis was completed using a random effect meta-analysis. A Woolf’s test of homogeneity of regression coefficients across batches was performed, i.e. the distribution of regression estimates across batches for each probe is compatible with that expected given a common regression estimate. All statistical tests were 2-sided, and analyses of individual batches were carried out using SAS (version 9.3; SAS Institute Inc., Cary, NC) and R (version 2.14.0). Meta-analyses were carried out using the R package *rmeta* (http://CRAN.R-project.org/package=rmeta). To control for multiple testing, associations with p < 5 × 10^-6^ were considered statistically significant (e.g., Bonferroni adjustment based on number of independent tests). Pathway analysis used Ingenuity Pathway Analysis (IPA) (Ingenuity® Systems, http://www.ingenuity.com) for genes closest to CpG probes associated with disease status or outcome at p < 0.0001.

The following linear model was used to determine if DNAm levels differ between EOC cases and matched controls for each CpG site. Let, ${Y_{i}}_{j}=\alpha_{j}+\beta_{j}X_{i}+\gamma_{j}^{T}Z_{i}+{e_{i}}_{j},$ where *Y*_*ij*_ represents the adjusted methylation beta value for subject *i* and CpG probe *j* (*j* = 1…, 13816), *X*_*i*_ represents disease status for subject *i* (1 if case and 0 if control), *Z*_*i*_ represents covariates for subject *i* and ${\varepsilon_{i}}_{j}\sim N\left(0,\sigma_{j}^{2}\right).$ To identify covariates that differ between EOC cases and controls to include in the model (i.e., potential confounders), potential covariates were examined for association with disease status within a stepwise logistic regression model, resulting in the inclusion of parity/age at first live birth combination (nulliparous, 1-2 and age < = 20 years, 1-2 and age > 20 years, 3+ and age < = 20 years, 3+ and age > =20 years, missing), current alcohol use (never, former, current, missing), current smoking status (never or former, current, missing), enrollment year, and recruitment state (MN vs. non-MN). For each CpG probe *j*, the disease status parameter ( $\hat{\beta_{j}}$) was estimated using the rank-transformed adjusted beta methylation values, along with a 95% confidence interval (CI).

We assessed associations of methylation beta values with overall survival (OS) using Cox proportional hazards regression analyses, adjusted for age at diagnosis, tumor stage (III/IV, I/ II), presence of ascites (yes, no, missing) and volume of residual tumor following debulking surgery (<1 cm, >1 cm, missing) based on stepwise Cox regression analysis. The proportionality assumption was assessed by the analysis of scaled Schoenfeld residuals for all covariates included in the statistical analysis and found to be upheld [18]. We accounted for left truncation using start-stop counting process style of input and estimated hazards ratios (HR) and 95% CIs [19].

## Results

### Disease status and DNA methylation

**Figure 1:**
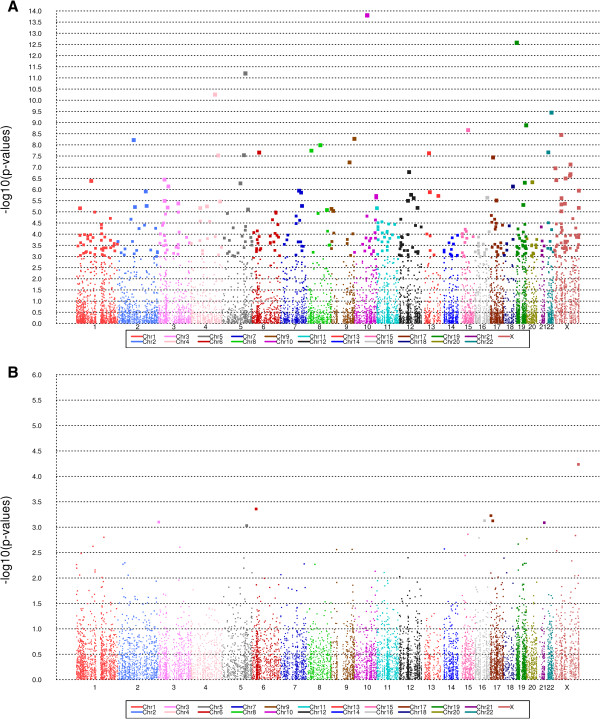
**Manhattan plots of the –log10(p-value) vs. CpG location. (A)** Association of CpGs and EOC status. Analysis adjusted for parity/age at first live birth combination, alcohol use, current smoking status, enrollment year, and recruitment state. **(B)** Association of CpGs and overall survival. Analysis adjusted for age at diagnosis, tumor stage, presence of ascites and volume of residual tumor following debulking surgery.

**Figure 2:**
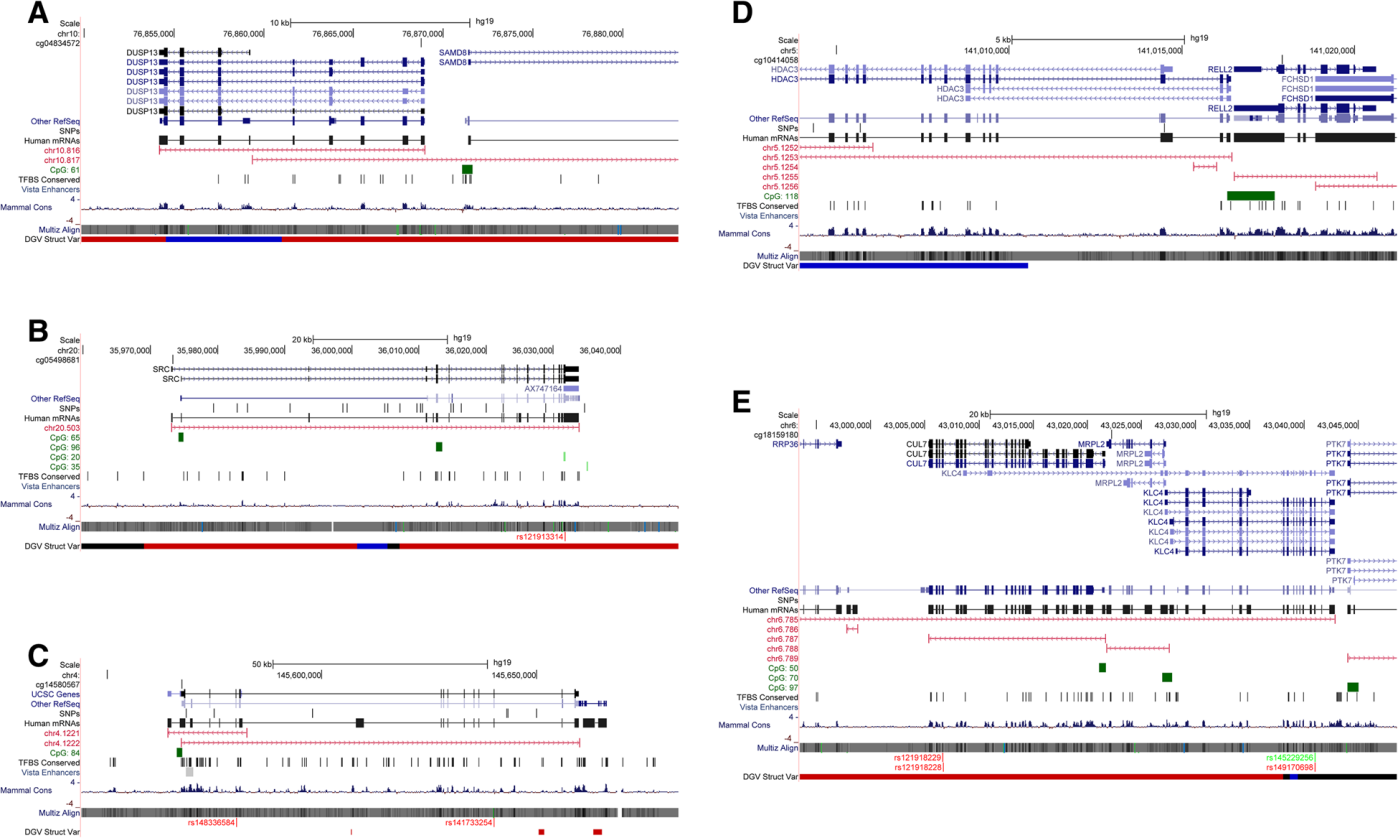
**Differential methylation regions between EOC cases and controls displayed in UCSC Genome Browser. (A)***DUSP13* region; **(B)***SRC* region; **(C)***HHIP* region; **(D)***HDAC3* region; and **(E)***CUL7* region.

In a meta-analysis across the three batches (two sets of experiments involving the Illumina Infinium HumanMethylation27 beadchip and one experiment involving the Illumina Infinium HumanMethylation450 beadchip) evaluating association between each of the 13,816 CpG probes and ovarian cancer case-control status (336 cases, 398 controls), 30 CpGs showed p-value ≤ 5×10^-7^ (Table 2), where none of the tests for heterogeneity of effects across batches were significant (p > 0.05). We confirmed that these 30 CpGs were also included in the Koestler et al. (2012) analysis, and thus determined not to be associated with cell type distribution. Of these CpGs, the following were also replicated in an independent study (p < 0.001) conducted by Teshendorff et al. [14]: cg04834572 near *DUSP13*, cg10414058 near *HDAC3*, cg19280776 near *PAG1*, and cg24959428 near *GBP6*. In addition to the replication of specific CpG sites, *C19orf18* and *MARCH1* contained CpG sites found to be replicated for association with EOC risk [20]. All CpG sites, with the exception of a CpG near *PAG1,* had negative parameter estimates indicating lower methylation in the cases as compared to controls (e.g., cases were hypo-methylated). Plots of the entire set of results for the 13,816 CpG sites (i.e., sites contained in both the 27K and 450K arrays, specific and not associated with cell type distribution) are presented in Figure 1A. The top association between methylation and disease status, which as also replicated, was observed for the CpG probe cg04834572 located approximately 315 kb upstream of *DUSP13* on chromosome 10 (Figure 2A) with a meta-analysis p-value of 1.6 × 10^-14^ and individual batch p-values ranging from 2.1×10^-4^ to 1.1 × 10^-6^. *DUSP13* is a member of the protein-tyrosine phosphatase superfamily and interacts with protein kinases involved in the regulation of cell proliferation and differentiation. Other significantly associated CpG sites were near biologically interesting/relevant genes, such as *SRC* (cg05498681; p = 4.8×10^-7^) (Figure 2B), *HHIP* (cg14580567; p =5.6×10^-11^) (Figure 2C), and replicated CpG near *HDAC3* (cg10414058; p = 6.3×10^-12^) (Figure 2D).

**Table 2:** **CpG sites associated with disease status (p ≤ 5×10**^**-7**^**)**

**Probe ID**	**Ch**	**Position (bp)**	**Nearest genes**	**Location of nearest gene (bp)**^**#**^	**Location to Island**^**^**^	**Meta-Analysis**^**§**^		**Batch 1**		**Batch 2**		**Batch 3**	
						$\hat{\beta }$	**P**	$\hat{\beta }$	**P**	$\hat{\beta }$	**P**	$\hat{\beta }$	**P**
cg04834572*	10	76868766	*DUSP13*	76854190-76868970	Shelf	-0.65	1.6E-14	-0.82	2.1E-4	-0.55	6.0E-6	-0.73	1.1E-6
cg11722531	19	1449857	*APC2*	1450148-1473243	Shore	-0.62	2.7E-13	-0.64	3.5E-3	-0.54	6.4E-6	-0.74	1.3E-6
cg10414058*	5	141017903	*RELL2*	141016517-	Shore	-0.94	6.3E-12	-1.13	3.5E-8	-0.71	7.1E-10	-1.05	9.0E-14
			*HDAC3*	141020631									
				141000443-141016423									
cg14580567	4	145567271	*HHIP*	145567148-145659881	Island	-0.56	5.6E-11	-0.49	0.024	-0.55	3.8E-6	-0.61	9.3E-5
cg08245789	22	40289538	*ENTHD1*	40139049-40289794		-0.56	3.6E-10	-0.66	4.0E-3	-0.43	3.9E-4	-0.69	6.5E-6
cg27623214	19	58485726	*C19orf18*^†^	58469805-58485902		-0.52	1.3E-9	-0.66	2.9E-3	-0.52	1.4E-5	-0.45	3.4E-3
cg23877385	15	59908652	*GCNT3*	59903982-59912210		-0.52	2.2E-9	-0.46	0.043	-0.53	1.3E-5	-0.52	6.3E-4
cg26150490	X	47863595	*SPACA5*	47863734-47869130	Island	-0.50	3.6E-9	-0.34	0.10	-0.54	6.8E-6	-0.52	6.6E-4
			*ZNF182*	47834250-47863394									
cg22336401	9	140336227	*ENTPD8*	140328816-140335901	Island	-0.50	5.3E-9	-0.27	0.24	-0.51	2.5E-5	-0.60	9.1E-5
cg20775254	2	95940705	*PROM2*	95940201-95957056		-0.53	6.1E-9	-0.58	5.3E-3	-0.41	8.2E-4	-0.69	5.6E-6
cg19280776*	8	82024586	*PAG1*	81880045-82024303	Shore	0.49	1.0E-8	0.60	6.2E-3	0.52	1.4E-5	0.38	0.013
cg07634191	8	27850178	*SCARA5*	27727399-27850369		-0.48	1.8E-8	-0.50	0.025	-0.39	1.2E-3	-0.61	4.5E-5
cg21244955	22	21192955	*PI4KA*	21061979-21213100		-0.49	2.2E-8	-0.47	0.039	-0.48	6.6E-5	-0.50	1.3E-3
cg18159180	6	43022213	*CUL7*	43005355-43021683	Shore	-0.48	2.2E-8	-0.35	0.12	-0.57	2.8E-6	-0.41	8.0E-3
			*MRPL2*	43021767-43027242									
cg04439215	13	45768901	*KCTD4*	45766988-45775175		-0.49	2.4E-8	-0.65	4.4E-3	-0.37	2.2E-3	-0.60	1.0E-4
			*GTF2F2*	45694631-45858240									
cg26787239	5	132008525	*IL4*	132009678-132018370		-0.48	2.9E-8	-0.50	0.025	-0.50	3.4E-5	-0.43	4.8E-3
cg07259382	4	164536228	*MARCH1*^†^	164445450-165304407		-0.48	3.0E-8	-0.58	8.5E-3	-0.43	3.1E-4	-0.50	1.2E-3
cg14808739	17	17741098	*SREBF1*	17714663-17740325	Shore	-0.48	3.7E-8	-0.41	0.063	-0.52	2.3E-5	-0.44	3.8E-3
cg00065385	9	111623395	*ACTL7A*	111624603-111626035		-0.47	6.1E-8	-0.50	0.026	-0.49	5.0E-5	-0.43	6.9E-3
cg04721883	X	103499577	*ESX1*	103494719-103499599	Island	-0.46	7.6E-8	-0.40	0.068	-0.38	1.7E-3	-0.62	4.6E-5
cg21400640	X	12992967	*TMSB4X*	12993226-12995346	Shore	-0.46	1.1E-7	-0.50	0.029	-0.46	1.4E-4	-0.44	4.0E-3
cg11871280	12	60082038	*SLC16A7*	59989821-60183636		-0.45	1.7E-7	-0.24	0.28	-0.51	2.4E-5	-0.46	3.1E-3
cg09261015	X	103499647	*ESX1*	103494719-103499599	Island	-0.45	2.1E-7	-0.46	0.046	-0.39	1.1E-3	-0.54	4.7E-4
cg18731813	X	100805683	*ARMCX1*	100805514-100809683	Shelf	-0.44	2.4E-7	-0.23	0.30	-0.46	1.3E-4	-0.51	7.3E-4
cg23279136	X	74375966	*ABCB7*	74273105-74376132		-0.44	3.2E-7	-0.62	6.3E-3	-0.33	5.7E-3	-0.54	4.9E-4
cg02254461	3	39195904	*CSRNP1*	39183342-39195102	Shore	-0.44	3.6E-7	-0.37	0.10	-0.48	9.1E-5	-0.42	6.1E-3
cg26246138	X	18372612	*SCML2*	18257433-18372844	Island	-0.44	3.8E-7	-0.37	0.096	-0.37	2.3E-3	-0.57	1.6E-4
cg24959428*	1	89829951	*GBP6*	89829436-89853719		-0.44	4.1E-7	-0.50	0.025	-0.42	5.2E-4	-0.44	4.4E-3
cg05498681	20	35973318	*SRC*	35973088-36033821	Shore	-0.44	4.8E-7	-0.17	0.45	-0.53	1.3E-5	-0.41	8.1E-3
cg01377911	19	49568036	*NTF4*	49564397-49567124		-0.43	5.0E-7	-0.38	0.086	-0.37	2.3E-3	-0.55	2.5E-4

^**#**^Locations based on NCBI (http://www.ncbi.nlm.nih.gov), build 37.

^Shore CpG sites defined to be within +/- 2 kb from CpG island; Shelf CpG sites defined to be within +/- 2kb of CpG Shore.

^**§**^All tests for heterogeneity of effects across the three batches were non-significant (p > 0.05); analysis adjusted for age at first live birth, alcohol use, smoking status, enrollment year, and recruitment state; a negative parameter estimate indicates lower methylation in the cases as compared to the control (e.g., cases where hypomethylated).

*Same CpG site found to be associated with EOC risk (p ≤ 0.001) in a prior report [14].

^†^CpG sites near this gene found to be associated with EOC risk in a prior report [14].

To identify any commonality of highlighted genes within biological pathways, pathway analysis using Ingenuity Pathway Analysis (IPA) was completed for the 155 genes closest to the CpG probes (based on Illumina provided annotation) that were associated with disease status based on a liberal threshold of p < 0.0001. The top pathways enriched for these 155 genes were the telomerase signaling (five genes in our top 155 were in the list of 99 genes within the telomerase signaling pathway; p = 1.24×10^-3^ for enrichment of pathway) and the paxillin signaling (five genes in our top 155 were in the list of 110 genes within the paxillin signaling pathway; p = 1.42×10^-3^). The five genes in the telomerase signaling pathway with methylation associated with disease status at p < 0.0001 were *HDAC3* (p = 6.33×10^-12^), *IL2RG* (p = 4.33×10^-6^), *PIK3C2B* (p = 1.97×10^-5^), *PIK3R1* (p = 5.19×10^-5^), and *POT1* (p = 1.38×10^-6^). *PIK3C2B* has been implicated in development of glioblastoma multiforme, while mutations in *PIK3R1* have been seen in ovarian tumors and cancer cell lines and endometrial cancer [21-23]. *POT1* has been found to be associated with tumor stage and telomere length in gastric cancer [24-26]. For the paxillin signaling pathway, the five differentially methylated CpGs were near *ARFIP2* (p = 4.60×10^-5^), *ITGB6* (p = 3.95×10^-5^), *PIK3C2B*, *PIK3R1* and *SRC*, with some overlap between the top two pathways (*PIK3R1* and *PIK3C2B*).

### Survival following EOC and DNA methylation

**Figure 3:**
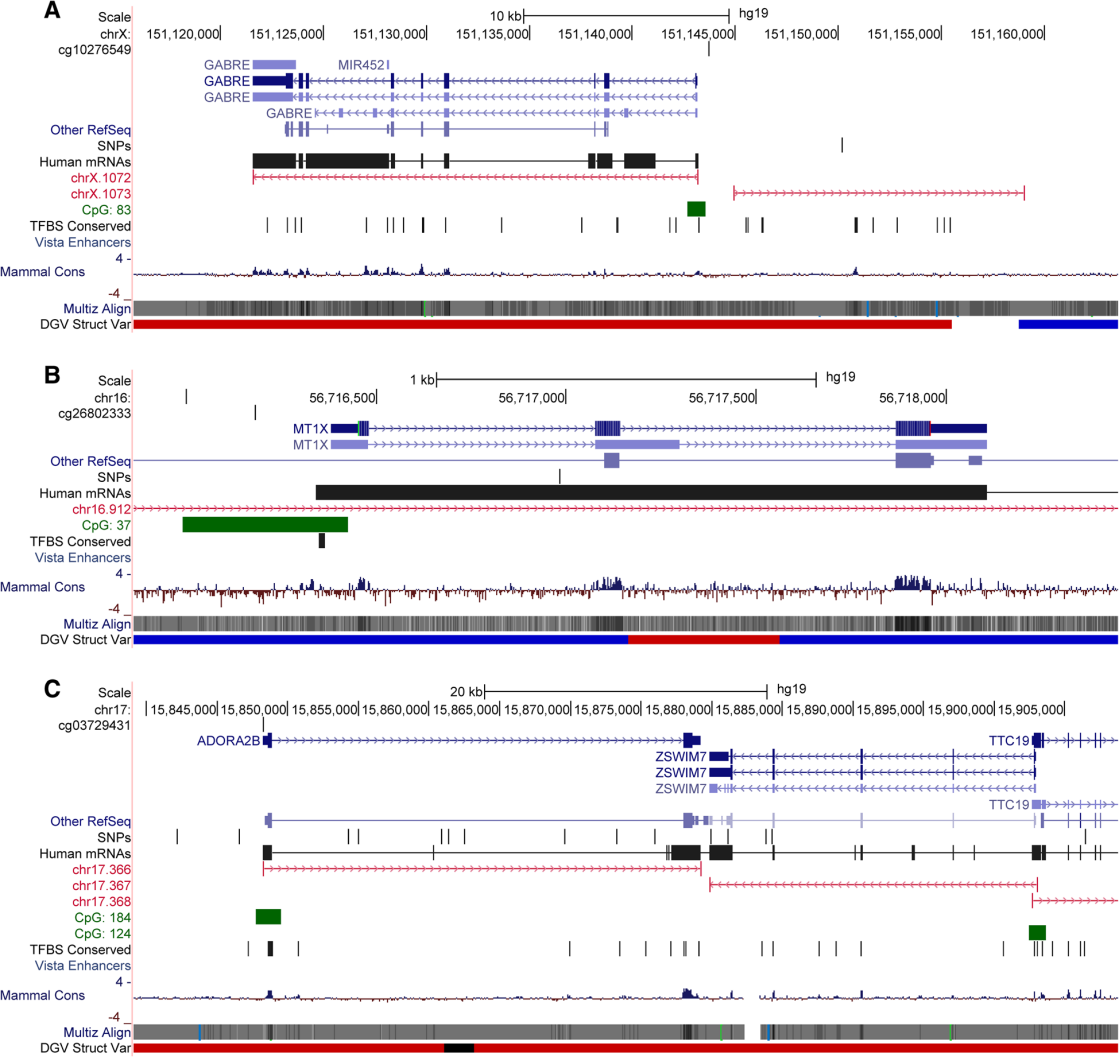
**Methylation regions associated with overall survival displayed in UCSC Genome Browser. (A)***GABRE* region; **(B)***MT1X* region; and **(C)***ADORA2B* region.

Many fewer CpGs were associated with OS among the 366 cases than with case-control status, as illustrated in Figure 1B. None of the associations were statistically significant at the 5×10^-6^ level; the top eight CpG probes with meta-analysis p-value < 10^-3^ for association with OS are presented in Table 2. The top CpG sites associated with OS were cg10276549 within the promoter region of *GABRE* (p = 5.8×10^-5^) (Figure 3A) and CpG site (cg06171242) within the promoter region of *TTRAP*/*TDP2* (p = 4.4×10^-4^). *GABRE* is a target for many benzodiazepine drugs used in the treatment of pain, insomnia, epilepsy, anxiety and panic related disorders [27-29]. However, little information can be found implicating a role of *GABRE* in response to chemotherapies (http://www.cancer.gov/clinicaltrials/). In addition to the modest level of association for CpGs near *GABRE*, there was a trend for association of CpG sites near the following biologically relevant genes: *MT1X* (p =7.4×10^-4^) (Figure 3B), *ADORA2B* (p = 7.4×10^-4^) (Figure 3C), and *ABLM3* (p = 9.3×10^-4^). These three CpG sites moderately associated with OS were all within CpG islands or shores and within the promoter region of the corresponding gene.

Similar to the analysis of the disease-associated genes, an exploratory pathway analysis using IPA was completed for the 61 genes closest to the CpG probes most associated with OS (meta-analysis p < 0.01). The top canonical pathways enriched for these 61 genes were relaxin signaling (five genes out of 147; *GNA12*, *GNB1*, *PIK3R4*, *RAP1A*, *TDP2*; p = 7.09×10^-5^ for enrichment of pathway) and CXCR4 signaling (five genes out of 160; *GNA12*, *GNB1*, *ITPR1*, *PIK3R4*, *ROCK1*; p = 1.25×10^-4^) and IL-8 signaling (five genes out of 192; *ARRB2*, *GNA12*, *GNB1*, *PIK3R4*, *ROCK1*; p = 3.05×10^-4^). Three genes (*GNB1* (p = 0.006), *GNA12* (p = 0.009), and *PIK3R4* (p = 0.002)) are part of all three of these canonical pathways.

## Discussion

Via a CpG-by-CpG approach excluding CpGs known to correlate with potentially confounding white blood cell types, we identified methylation CpG sites (and corresponding neighboring genes) with differential specific hyper- or hypo-methylation signals by case-control status and by survival time. To increase power to detect differentially methylated CpG sites, we completed a meta-analysis of results from three DNAm experiments using two genome-wide methylation arrays, restricting focus to high quality probes on both arrays.

A number of CpG sites were found to be differentially methylated between EOC cases and age-matched controls (Table 2). The CpG site that was most differentially methylated between EOC cases and controls was cg04834572 located at the splice region of exon 1 and intron 1 of *DUSP13* (p = 1.6×10^-14^). The blood-based methylation of this CpG site was also reported to be associated with EOC risk in a previous study with p = 0.002 (Figure 2A) [14]. In addition to the replication of the association for the methylation site at *DUSP13*, four regions identified in this study were replicated for association EOC risk with a p ≤ 0.001, as reported in Teschendorff et al. [14]: cg02449608 (*C19orf18*, p = 0.0002), cg19280776 (*PAG1*, p = 8×10^-6^); cg17271365 (*MARCH1*, p = 2×10^-5^), cg10414058 (*HDAC3*, p = 0.001), and cg24959428 (*GBP6*, p = 0.001).

Many of the genes neighboring the top associated CpG sites have biological relevance to cancer development. Methylation at a CpG site on chromosome 20 at bp 35973318 (cg0549868), located within the splicing region of exon 1 and intron 1 of gene *SRC* (35973088-36033821 bp), was found to be associated with EOC risk (p = 4.8×10^-7^) (Figure 2B). *SRC* is a proto-oncogene which regulates EGFR, Akt, MAPK1 and NF-κB. SRC is a target for many anticancer drugs [30]. A CpG island (cg14580567, bp 145567271) within *HHIP* (145567148-145659881 bp) was also found to be associated with EOC risk (p = 5.6×10^-11^). The genomic region surrounding *HHIP* (hedgehog-interacting protein) (Figure 2C) has been implicated in many cancers, with hypermethylation of the promoter region found to down-regulate the expression of HHIP found in many tumors, such as gastric and pancreatic cancer [31]. The hedgehog proteins are evolutionarily conserved and are important for a wide range of developmental processes; members of this family control cell proliferation and differentiation, thus linking them with many cancers, including basal-cell carcinoma, small cell lung cancer and pancreatic cancer [32].

The methylation at a CpG site in the shore of a CpG island, approximately 2.5 kb upstream of *HDAC3,* was observed to be associated with EOC risk (p =6.3×10^-13^) (Figure 2D). This association was also observed in a previous study (p = 0.001) [14]. Other studies looking at the role of histone deacetylases (HDACs) found that the expression of HDAC1, along with the expression levels of HDAC2 and HDAC3, to be increased in ovarian tumors compared to levels in benign tumors and normal tissue, suggesting the oncogenic potential of HDACs in ovarian tumors [33-35]. Lastly, a CpG site near *CUL7 (*cg18159180, p = 2.2×10^-8^) was differentially methylated between EOC cases and controls; *CUL7* has been shown to block Myc-induced apoptosis in a p53-dependent manner (Figure 2E) [36,37].

In addition to individual CpG sites associated with EOC risk, we also found the telomerase signaling and paxillin signaling pathways to be enriched for genes with CpGs that were differentially methylated between cases and controls. The telomerase signaling pathway and inherited variation in *TERT* have been found to be associated with the development of EOC and other cancers [11]. The maintenance of functional telomeres is critical in that telomeres that become too short are unable to protect the chromosome from DNA damage. TERT plays an extensive role in the maintenance of functional telomeres, and TERT can be activated by AKT and HSP90 and inhibited by c-Abl. One gene identified to be moderately associated with EOC risk was *PIK3R1,* which is also a member of the telomerase signaling pathway. *PIK3R1* is involved in ATPase and estrogen receptor binding and regulates numerous genes, such as *AKT, NFKB, TNF,* and is involved in apoptosis, proliferation and differentiation. *PIK3R1* has also been linked to epithelial neoplasia and cancer, endometrial ovarian cancer, and endometrioid carcinoma [20,22,23,38]. In contrast, the paxillin signaling pathway is involved in the recruitment of signaling and structural proteins to paxillin required for regulation of cell motility, with many of the paxillin-binding proteins having oncogenic equivalents.

In contrast to the findings for EOC risk, we found no statistically significant CpG probes associated with OS following EOC (Table 3). However, many of genes surrounding these CpG sites have potential biological relevance and would be warranted for future follow-up. In particular, the gene *GABRE (*Figure 3A) is a target for many benzodiazepine agents [27,28]; *MT1X (*Figure 3B) has been implicated in resistance to cisplatin therapy in oral squamous cell carcinoma and irinotecan resistance in gastric cancer patients [39,40]; *ADORA2B (*Figure 3C) is an antagonist in many drugs (such as dyphylline and aminophylline, used in treatment of asthma and pulmonary emphysema), with recent research discovering antagonists of *ADORA2B* are preferentially toxic to breast tumor cells expressing Fra-1, a candidate metastasis gene and expression of *ADORA2B* up-regulated in colorectal carcinoma tissues and cell lines [41,42].

**Table 3:** **CpG sites associated with overall survival following EOC (p < 10**^**-3**^**)**

**Probe**	**Ch**	**Position (bp)**	**Nearest genes**	**Location of nearest gene (bp)**^**#**^	**Relation to Island**^**^**^	Meta-Analysis*		**Batch 1**		**Batch 2**		**Batch 3**	
						**HR**	**P**	**HR**	**P**	**HR**	**P**	**HR**	**P**
cg10276549	X	151143686	*GABRE*	151121596-151143151	Shore	0.95	5.8E-5	0.96	0.32	0.97	0.19	0.94	2.9E-4
cg06171242	6	24667490	*ACOT13*	24667263-24705297	Shore	1.10	4.4E-4	1.20	0.22	1.11	7.2E-3	1.02	0.87
			*TTRAP/TDP2*	24650205-24667115									
cg14360897	17	4843676	*RNF167*	4843630-4848517	Shore	1.22	5.9E-4	1.25	0.084	1.29	3.8E-3	1.13	0.20
			*SLC25A11*	4840425-4843462									
cg26802333	16	56716182	*MT1X*	56716382-56718108	Island	1.07	7.4E-4	1.11	0.044	1.07	1.2E-3	1.00	0.97
cg03729431	17	15848264	*ADORA2B*	15848231-15879210	Island	1.11	7.5E-4	0.97	0.85	1.12	5.8E-3	1.09	0.69
cg21858376	3	4534791	*ITPR1*	4535032-4889524	Island	1.12	7.9E-4	1.31	0.11	1.11	0.022	1.09	0.37
cg12003230	21	44899139	*C21orf84/*	44881974-44898103		0.86	8.1E-4	0.82	0.022	0.85	8.9E-3	0.97	0.72
			*LINC00313*										
cg05026186	5	148520876	*ABLIM3*	148521054-148639999	Shore	0.96	9.3E-4	0.98	0.41	0.96	5.3E-3	0.96	0.071

^**#**^Locations based on NCBI (http://www.ncbi.nlm.nih.gov), build 37.

^Shore CpG sites defined to be within +/- 2 kb from CpG island; Shelf CpG sites defined to be within +/- 2kb of CpG Shore.

*All tests for heterogeneity of effects across the three batches were non-significant (p > 0.05); analysis adjusted for age at diagnosis, tumor stage, presence of ascites and volume of residual tumor following debulking surgery.

Single CpG probe analysis of the association of blood-based DNAm with survival following EOC, followed by pathway analyses found the top pathways to all contain three genes (*GNB1, GNA12*, and *PIK3R4),* although individual CpG evidence for these three genes were modest*.* The standard chemotherapy regimen for EOC patients following surgery is a combination therapy involving a taxane (e.g., paclitaxel) and platinum (e.g., cisplatin, carboplatin) agent, increasing our interest in the gene *GNA12* found to be associated with response to cisplatin/paclitaxel [43]. Guanine nucleotide binding protein (G protein), beta polypeptide 1 (GNB1) has been recently found to be associated with breast cancer outcomes and clinical and pathological measurements [44]. *PIK3R4* is a member of the phosphoinositide 3-kinases (PI3Ks) family that is involved in multiple cell functions (e.g., proliferation, cell survival, degranulation), and this gene is a novel candidate for outcome following EOC.

In summary, we have identified several methylation CpGs sites, using blood-based or leukocyte DNAm, which are differentially methylated by case-control status. Of these CpGs, four CpGs and two genes containing significant CpGs were replicated in an independent study of DNAm and EOC risk. Strengths of our study are large sample size, exclusion of CpGs associated with white blood cell types, and inclusion of relevant covariates. Prior work in a smaller set of cases and controls showed that blood-based DNAm associated with case-control status [14], thus providing additional evidence to “confirmed” CpG regions associated with EOC. To ensure that none of our findings could be attributed to confounding due to cell type distribution, we removed of probes associating with cell types (which in fact showed very strong associations with case-control status; data not shown). In addition to these strengths, there are also limitations to this study. First, this study was limited to CpG sites assayed on the Illumina array; future application of genome-wide DNA methylation sequencing (i.e., methyl-seq) will enable additional EOC related methylation marks to be discovered. Secondly, the retrospective case-control design used in this study precludes interpretation of these results as indicators of EOC risk. As blood was drawn upon diagnosis, we cannot exclude the possibility that the case-control differences resulted from the cancer itself, from its treatment, or from lifestyle changes. Nonetheless, this short list of CpGs should be of high priority for cohort studies with baseline blood draws and follow-up for later EOC. We note that our survival studies were limited primarily by sample size (336 cases), and thus may have been underpowered to detect modest effects; combining this study with other blood-based methylation case studies will be a key next step.

## Conclusion

In conclusion, this early examination of blood-based DNAm provides added experience to a relatively nascent field, suggesting that careful pre-processing and consideration of probes associating with distributions of white blood cell types is critical. We also report specific CpGs that associate either with case-control status or outcome, which are worthy of follow-up in prospective cohort and clinical studies.

## Competing interest

The authors declare that they have no conflict of interest.

## Authors’ contributions

ELG, JMC, BLF, JEO, KRK, and MSC participated in the design of the study and coordination. MSC, KRK, VS, JMC prepared the samples and completed the assays for measuring DNA methylation. BLF, SMA, MCL carried out the statistical analyses included the manuscript. DNR provided the annotation of the regions and CpGs. CW, SW and DCK were involved in the quality control and normalization of the DNA methylation array data, in addition to BLF, SMA and MCL. BLF and ELG drafted the manuscript. All authors read and approved the final manuscript.

## Pre-publication history

The pre-publication history for this paper can be accessed here:

http://www.biomedcentral.com/1755-8794/7/21/prepub

## Supplementary Material

Additional file 1: Figure S1 Plot of the 1^st^ and 2^nd^ principal components for each of the three batches before and after the normalization step. The different colors in the figures represent the different plates of 96 samples in each batch. Batch 1 Pre **(A) **and Post **(B)** adjustment; Batch 2 Pre **(C)** and Post **(D)** adjustment; Batch 3 Pre **(E)** and Post **(F)** adjustment. Click here for file
