# Supplementary figures and images for: Methylation of leukocyte DNA and ovarian cancer: relationships with disease status and outcome
Source: BMC Med Genomics. 2014 Apr 28;7:21. doi: 10.1186/1755-8794-7-21 (PMC4102255; doi:10.1186/1755-8794-7-21)

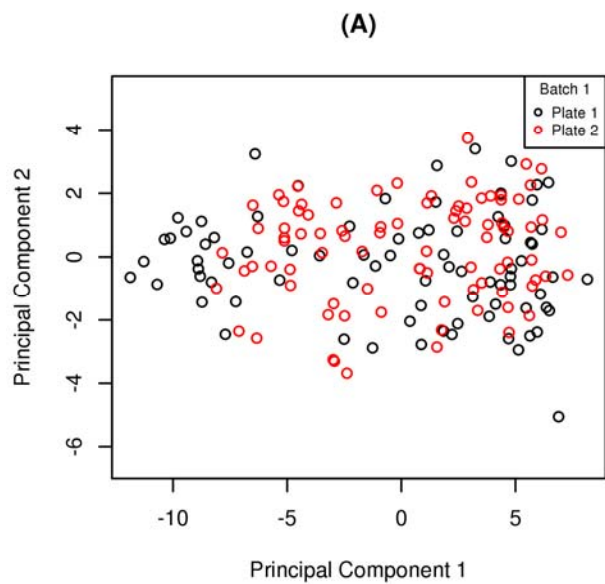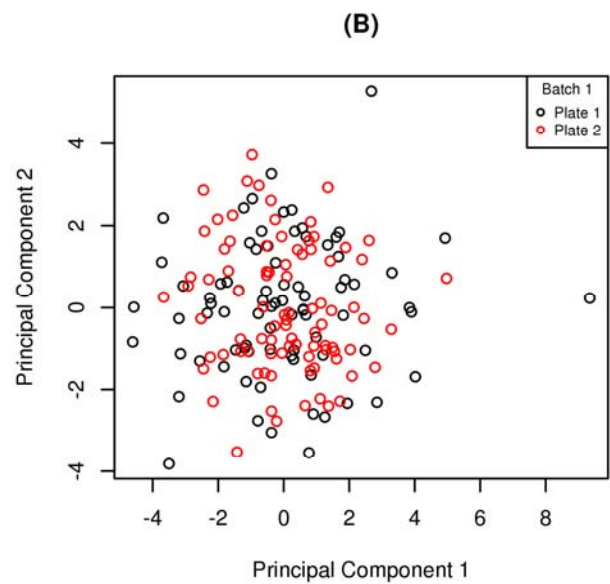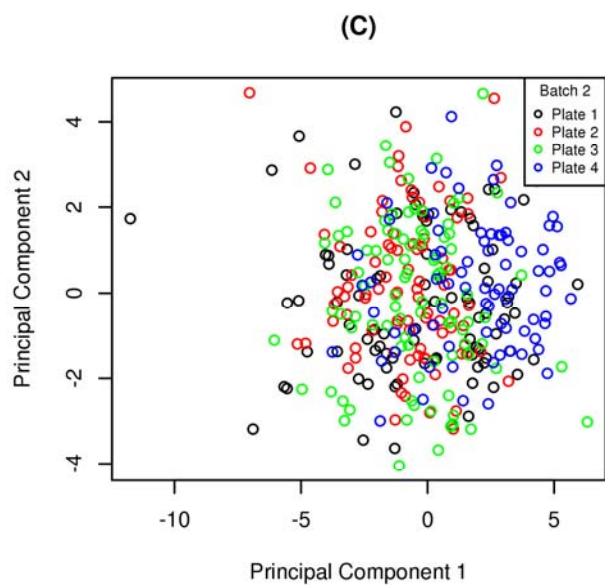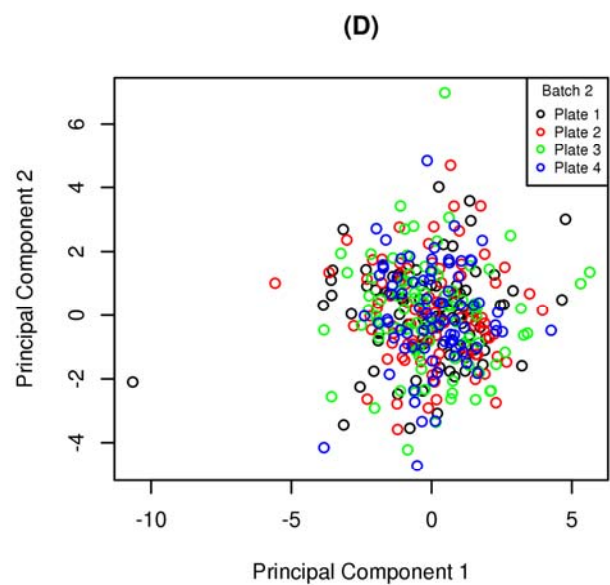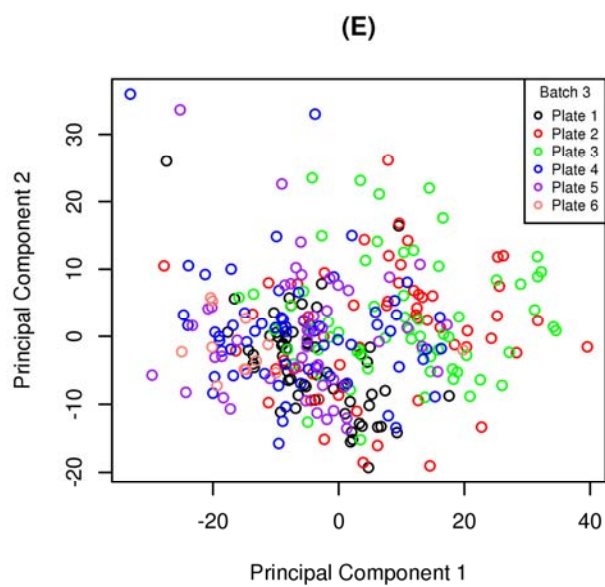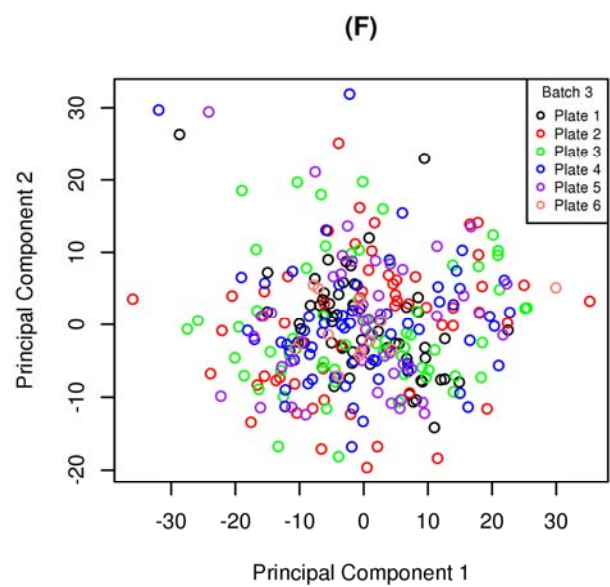

Supplement: Additional file 1: Figure S1 — Plot of the 1st and 2nd principal components for each of the three batches before and after the normalization step. The different colors in the figures represent the different plates of 96 samples in each batch. Batch 1 Pre (A) and Post (B) adjustment; Batch 2 Pre (C) and Post (D) adjustment; Batch 3 Pre (E) and Post (F) adjustment. [file 1755-8794-7-21-S1.pdf]
